# Vaccine for Covid-19 and Pregnant Women

**DOI:** 10.1055/s-0041-1731382

**Published:** 2021-07-27

**Authors:** Beuy Joob, Viroj Wiwanitkit

**Affiliations:** 1 Private Consultant, KMT, Bangkok, Thailand; 2 Department of Community Medicine, DY Patil University, Pune, Maharashtra, India

Dear Editor,

We would like to share ideas on “We have Vaccine for Covid-19! What to Recommend for Pregnant Women?” ^1^ Quintana ^1^ concluded that “ *The final decision whether or not to receive the vaccine will be made by the woman after receiving the appropriate information. This same principle applies with even greater emphasis to puerperal and lactating women* .” ^1^ Indeed, the Covid-19 vaccine is considered risky for any subject. As a vaccine with an emergency-use authorization, the data on its safety and efficacy against COVID-19 is insufficient. According to a recent report on the joint International Federation of Fertility Societies (IFFS)/ European Society of Human Reproduction and Embryology (ESHRE) statement on COVID-19 vaccination for pregnant women, Ory et al. ^2^ concluded that “ *individual risk, availability of the vaccine, and the potential recipients' concerns regarding unknown risks of the new vaccines* ” should be important factors for considering and deciding to receive or not receive the vaccine. An important question is whether a pregnant woman has or not a higher risk of developing adverse effects than the general population. If a woman of childbearing age can bee vaccinated, there should be no increased risk for the mother. The remaining question to be researched is regarding the possibility that the immunity can cross the placenta and affect the fetus. Finally, while we wait for more data on the vaccine, there should be a careful reanalysis of the actual risk of pregnant subjects developing infections and a severe disease if infected. This is an interesting issue for further research in obstetrics.

**Conflict of Interests**

The authors have no conflict of interests to declare.

**References**

1. Quintana SM. We have vaccine for COVID-19! What to recommend for pregnant women? Rev Bras Ginecol Obstet 2021;43(2):81–83 10.1055/s-0041-1726090

2. Ory S, Veiga A, Horton M, Gianaroli L. Joint IFFS/ESHRE statement on COVID-19 vaccination for pregnant women and those considering pregnancy. Hum Reprod Open 2021;2021(2):hoab016

**Author's Reply**

^1^ Faculdade de Medicina de Ribeirão Preto, Universidade de São Paulo, Ribeirão Preto, SP, Brazil

Silvana Maria Quintana, Avenida Bandeirantes 3.900, Ribeirão Preto, São Paulo, SP, 14049-900, Brazil (e-mail: quintana@fmrp.usp.br).

## Reply to “Vaccine for COVID-19 and pregnant women”

**Silvana Maria Quintana ^1^** 0000-0002-9311-786X

Dear Editor,

Thank you for the author's comments, and I am very pleased to share ideas on such an important topic as the prevention of Covid-19 infection in pregnant women through vaccines. The editorial reflects my opinion based on the studies available so far; however, I emphasize that the infection is recent, and we are learning on a daily basis. In Brazil, we have experienced a significant increase in maternal deaths associated with Covid-19, according to official data from the Brazilian Ministry of Healthl ( https://observatorioobstetrico.shinyapps.io/covid_gesta_puerp_br/ ). This situation has brought great concern and the urgent need to reduce maternal deaths due to Covid-19. Although the studies on the Covid-19 vaccine did not include pregnant women and puerperal women in their designs, available data have shown that vaccinated pregnant women do not develop more serious or more frequent adverse events when receiving a vaccine compared with a non-pregnant population. Obviously, pregnant women and women who have recently given birth should be advised about the limited data on the immunogenicity and safety of the Covid-19 vaccines before deciding to get vaccinated or not. Given this context, the Brazilian Ministry of Health has released a vaccine calendar for all pregnant women in two stages. In the first phase, only pregnant women with comorbidities will receive the vaccine, and, in the second phase, all pregnant women will be vaccinated. I hope that pregnant women will have been vaccinated soon in order to reduce the maternal deaths from this infection in Brazil.

